# Intraoperative radiotherapy for the treatment of thyroid cancer: a pilot study

**DOI:** 10.18632/oncotarget.12901

**Published:** 2016-10-25

**Authors:** Pei-Qiang Yi, Fang-Fang Nie, You-Ben Fan, Wei-Wei Yu, Chao-Su Hu, Xiao-Mao Guo, Jie Fu

**Affiliations:** ^1^ Department of Radiation Oncology, Shanghai Jiaotong University Affiliated Sixth People’s Hospital, Shanghai, China; ^2^ Department of Radiation Oncology, Fudan University Shanghai Cancer Center, Shanghai, China; ^3^ Department of Oncology, Shanghai Medical College, Shanghai, China; ^4^ Shanghai Medical College, Fudan University, Shanghai, China; ^5^ Department of Surgery, Shanghai Jiaotong University Affiliated Sixth People’s Hospital, Shanghai, China

**Keywords:** thyroid cancer, intraoperative radiotherapy, pilot study, radiation therapy, adverse reactions

## Abstract

We preliminarily evaluated the clinical feasibility and efficacy of intraoperative radiotherapy in patients with thyroid carcinoma. Nine thyroid cancer patients received intraoperative radiotherapy using an Intrabeam system. The dose was 3-4 Gy and the irradiation time ranged from 1 min 32 s to 7 min 33s. One case was a primary thyroid carcinoma, while the other cases were recurrent disease. Adverse effects, recurrence and survival were analyzed. In one patient, poorly differentiated thyroid carcinoma recurred 5 months after treatment, one patient developed a postoperative tracheal skin fistula, and one patient developed a wound infection. Because the affected areas were treated with both surgical resection and then radiotherapy, it is difficult to know which of those led to the adverse effects. Nonetheless, our results indicate that intraoperative radiotherapy can relieve the symptoms associated with thyroid cancer and improve the quality of life for these patients. It thus appears feasible to treat thyroid cancer patients with intraoperative radiotherapy.

## INTRODUCTION

New cases of thyroid carcinoma account for 1%-5% in all new cancers each year. Thyroid papillary carcinoma and follicular carcinoma account for more than 90% of thyroid cancers, while medullary thyroid carcinoma and undifferentiated carcinoma account for about 1.7% and 0.8%, respectively. Although the proportion of undifferentiated carcinomas is the smallest, its prognosis is the worst (1975-2012 in US; all races) [1]. For well-differentiated thyroid carcinoma with minimal residual disease after surgical resection and iodine 131 treatment (if necessary), the 5-year survival rate is close to 100%. Poorly differentiated thyroid carcinoma showed little iodine 131 intake, which made postoperative recurrence more likely. The 5-year survival rates for poorly differentiated and undifferentiated thyroid carcinomas were 70% and 0%, respectively [2]. Postoperative radiotherapy can significantly improve the 5-year survival rate for thyroid cancer patients [3], including those with poorly differentiated or undifferentiated types [4]; however the dosage of external irradiation is limited by the need to limit exposure of important organs such as the spinal cord, trachea, esophagus and jugular vein. With intraoperative radiotherapy (IORT), the surrounding normal tissue can be protected, enabling delivery of larger radiation doses to the residual tumor. Previous studies have affirmed the efficacy of IORT in breast cancer, skull base tumors, cervical cancer, oral cancer, rectal cancer and gastrointestinal cancer [5–14]. In this pilot study, we examined the efficacy of IORT in patients with advanced thyroid cancer.

## MATERIALS AND METHODS

The Intrabeam system uses low energy X rays that rapidly attenuate, which helps to protect surrounding tissue. This system has the advantages of being small, light weight, and ease to move. In addition, little radiation protection is needed for the operating room. This makes the system an ideal choice for thyroid cancer patients receiving IORT.

From July 2014 to September 2015, nine patients with thyroid carcinoma underwent IORT using the Intrabeam system. Prior to the operation, the diagnosis was confirmed through pathological examination, CT and MRI. The mean age of the patients was 55 years (range: 30-80 years) and included four females and five males. One case was primary poorly differentiated thyroid carcinoma, while the other eight were recurrent patients who had undergone palliative surgery. These eight cases included four cases of papillary thyroid carcinoma, three cases of poorly differentiated thyroid carcinoma, and one case of follicular thyroid carcinoma. The patient with primary poorly differentiated cancer received no surgery or I ^131^ therapy prior to the study, whereas the other eight patients had tumor resections (3 palliative resections, 5 gross resections). Only one patient, with poorly differentiated cancer, had no metastasis; the other eight patients had differing degrees of metastasis and invasion. These included: 1) lymph node metastasis and invasion to the surrounding muscle; 2) cervical lymph node metastasis and invasion of the skin, trachea and left recurrent laryngeal nerve; 3) pulmonary metastasis with tracheal compression; 4) tracheal and right internal jugular vein metastasis and invasion of the esophageal muscular layer; 5) pulmonary and right cervical lymph node metastasis with local infiltration between the trachea and throat; 6) sternal metastasis and possible tracheal invasion between the trachea and esophagus; 7) metastasis of the lung, brachiocephalic vessels, and right supraclavicular and brachiocephalic lymph nodes and invasion of the trachea, subclavian artery and right common carotid artery; and 8) cervical lymph node and lung metastasis with invasion of the trachea, throat and left carotid artery, and upper esophageal compression. The clinical classification according to the AJCC classification system [15–16] and treatment of all patients are shown in Table 1.

**Table 1:** Clinical classification and treatment of all patients

Case	Pathological results	Classification	Distant metastasis	XRS	Dose	Applicator Size (cm)	Treatment time	Follow up (mo)
1	Papillary carcinoma	pTxN1bM0 IVA	-	50KV	4Gy	2.0	1:32	22
2	Poorly differentiated carcinoma	pT4bN0M0 IVB	-	50KV	4Gy	4.0	5:24	21
3	Poorly differentiated carcinoma	pT4aN1aM0 IVA	-	50KV	4Gy	5.0	7:33	-
4	Poorly differentiated carcinoma	pT4N0M1 IVC	lung	50KV	4Gy	3.0	3:16	-
5	Follicular carcinoma	pT4aN0M0 IVA	-	50KV	4Gy	2.0	1:32	17
6	Papillary carcinoma	pT4aN1M1 IVC	lung	50KV	4Gy	2.0	1:33	17
7	Papillary carcinoma	pT4bN0M1 IVC	sternum, lung	50KV	3Gy	4.0	4:12	16
8	Poorly differentiated carcinoma	pT4bN1bM0 IVB	-	50KV	4Gy	4.0	5:31	16
9	Papillary carcinoma	T4bN1bM1 IVC	lung	50KV	4Gy	3.0	3:19	14

During the surgery, the tumors were resected, and surgeons were careful to protect normal tissues. The eight patients with non-primary thyroid cancer received functional neck lymph node dissection. The patient with right vocal cord paralysis caused by invasion of the trachea and left recurrent laryngeal nerve, which was vulnerable to injury during the operation, received a tracheotomy in case of suffocation. The other six patients also had varying degrees of tracheal compression or invasion, but did not receive a tracheotomy or tracheal resection. After the tumor tissue was removed, the tumor bed was fully exposed, and the protection of the surrounding normal tissues was noted. The applicator was placed in the target area, the 3-4 Gy IORT was delivered to the tumor bed using 50 KV XRS. The median dose was 4 Gy, and the mean dose was 3.9 Gy. The application device was a flat plate, 2-5 cm in size (average: 3.2 cm). Radiotherapy duration ranged from 1 min 32 s to 7 min 33 s (average: 3 min 45 s). The patient with primary poorly differentiated cancer without metastasis also received external irradiation after IORT during his hospitalization. One patient with poorly differentiated thyroid carcinoma had a recurrence 5 months later and received external irradiation at that time. Three patients received iodine 131 after surgery. The remaining patients received no additional antitumor treatment during the follow-up period. The follow-up period ranged from 14-22 months (mean: 17.6 months), and 2 patients were lost to follow-up. Sites of recurrence and irradiation are shown in Table 2.

**Table 2:** Recurrence site and irradiation sites

Case	Irradiation sites	Recurrent site
1	Tumor bed	-
2	Tumor bed	Neck
3	Tumor bed	Lost
4	Tumor bed	Lost
5	Between the trachea and esophagus	-
6	Tumor bed	-
7	Front of trache	-
8	Tumor bed	-
9	Tumor bed	-

## RESULTS

**Figure 1:**
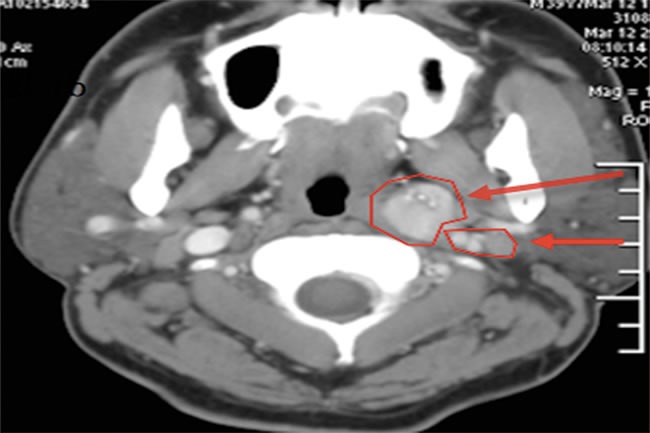
Preoperative CT scan of the neck Left thyroid mass (long arrow). Multiple enhanced lymph nodes were found in the periphery of the thyroid gland and in the left carotid sheath (short arrow).

**Figure 2:**
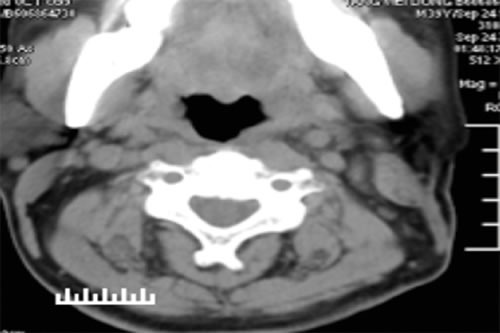
CT scan of the neck 6 months after surgery Note the absence of the left cervical mass and abnormal lymph nodes seen prior to treatment.

CT scans of the neck performed before and after the surgery and IORT show the beneficial structural effects of the treatment (Figures 1 and 2). Other beneficial effects of IORT were relief symptoms (tracheal compression) and impoved quality of life. One patient with poorly differentiated thyroid carcinoma relapsed after 5 months, one developed a postoperative tracheal skin fistula, and one developed wound infection. No other adverse effects were not found. Five patients experienced mild nausea and vomiting on the first day after surgery. Because patients received both surgical resection and radiotherapy, it is difficult to know whether the surgery or the radiotherapy led to the reported adverse effects.

## DISCUSSION

The 5-year survival rate for patients with well differentiated thyroid cancer is 98.2% [1], whereas the 5-year survival rates for poorly differentiated and undifferentiated thyroid cancers are 70% and close to 0%, respectively. Lennard et al [17] followed up 96 thyroid cancer patients for 50 years and reported that the total recurrence rate was 14%, the local recurrence rate was 13%, and the distant metastasis rate was 6%. Shen et al [18] reported that the total recurrence rate among 295 papillary thyroid carcinoma patients was 12.5%. Data from a phase three clinical trial on the drug treatment to thyroid cancer published in the NEJM [19] showed median progression-free survival to be 18.7 months in patients treated with lenvatinib and 3.6 months in patients receiving placebo. In our study, only one patient developed neck metastasis 5 months after treatment. This suggests IORT may be effective for controlling thyroid cancer recurrence.

Recurrent or poorly differentiated tumors often require external radiation therapy. External irradiation can significantly improve the 5-year survival rate in thyroid cancer patients, and reduce the rate of local recurrence of poorly differentiated thyroid carcinomas. External radiation therapy is also necessary for patients who had partial resection and postoperative recurrence [20]. Indeed, external irradiation was beneficial for thyroid cancer patients, no matter the pathological type, operation type, and recurrence status. The postoperative 5-year survival rate for thyroid cancer increased from 78% to 94% with application of radiation (50 Gy) to the surgical area [3]. However, the surrounding vital organs, including the spinal cord, parotid gland and larynx, limits the dose of external irradiation. Using IORT, a higher radiation dose can be applied to the residual tumor and draining lymph nodes while protecting the surrounding normal tissue.

The Intrabeam system uses low energy X-rays, which result in fewer adverse reactions with accurate positioning and skin protection. The biological effect of IORT was equivalent to 1.5-2.5 times that achieved with external irradiation. The nine patients in the present study received an average dose of 3.9 Gy (the highest was 4 Gy). The dose was selected based on reported IORT dosages used to treat breast cancer [21] and rectal cancer [6], and on our own experience. Peripheral nerves have the lowest tolerance (15 Gy), so 4 Gy (equal to 6-10 Gy of external irradiation) was considered safe. The average treatment time of 3 min 45 s caused no substantial extension of the operation.

In breast cancers, IORT resulted in less radiation toxicity and greater local tumor control than was achieved with external irradiation [5]. IORT was also used to safely treat locally advanced and recurrent rectal cancer, producing effects similar other intraoperative radiotherapy techniques but with a shorter operative time [6]. In addition, Weil et al [7] reported the feasibility of using IORT to treat brain metastases, and Wenz et al [22] reportedly used IORT to treat advanced thoracic spinal metastases, with good results. Finally, Rutkowski et al [9] reported the feasibility using IORT to treat early oral cancer.

Intraoperative electron radiation therapy (IOERT) has also been used to treat breast cancer. In a phase three clinical trial [10], the rate of ipsilateral breast tumor recurrence in the IOERT group was higher than in the external irradiation group, the overall survival rate did not differ between the two groups. However, in another phase three clinical trial, IOERT showed no advantage in local control and survival over surgery alone, but the security and reliability of IORET were confirmed [8]. For children with soft tissue sarcoma, IOERT can bring better local control with fewer adverse reactions at a lower dose than external radiation [12]. IORET also improves the control and survival rates for skull base tumors [13], and improves local control in advanced cervical cancer [14]. Calvo et al [8] reviewed the use of IOERT for gastrointestinal tumors, and suggested that IOERT improves local control of gastrointestinal cancer.

The GEC-ESTRO and ASTRO studies suggested that doctors should carefully choose IORT candidates. In addition, Wei et al [23] reported that an important contraindication of IORT is metastasis or tumor that is widely spread. But Gunderson et al [24] reported that even in cases of distant metastases or post-peritoneal implantation metastasis, treatment may be indicated under the following conditions: 1) resectable single organ metastasis; 2) excellent systemic treatment; 3) systemic disease with slow progression. In our report, eight patients had varying degrees of metastasis, but the large lesion in their necks was sufficient to compress the respiratory tract and seriously reduce their quality of life. These patients were therefore recommended for intraoperative radiotherapy. We think the management was appropriate for these patients.

One patient was implanted with titanium mesh in a previous operation, and their adverse reactions (tracheal skin fistula) was likely due mainly to the mesh. Postoperative wound infection occurred in one patient, and five patients experienced mild nausea and vomiting on the first day after operation. This was mainly due to the anesthesia. Patients received surgery and then IORT, making it is difficult to selectively determine the relation between surgery or radiotherapy and tracheal fistula, wound infection or other adverse effect.

Only a single article on the use of IORT or IOERT to treat thyroid cancer was retrieved on PubMed [25]. Those investigators reported five cases in1995, and we have now added nine more. We hope that our findings spur further research into the efficacy of IORT in the treatment of thyroid cancer.

Our study has several limitations. First, the number of cases is small, and some of the patients had recurrent disease or received palliative surgery, which makes the patients situation complex. Second, the clinical classification and differences between poorly differentiated carcinoma and undifferentiated carcinoma remains controversial. The AJCC classification criteria do not describe poorly differentiated types, so we regarded poorly differentiated types as undifferentiated carcinoma. Third, the follow-up time was short.

As of 2015, there have been a total of 180 clinical trials of IORT, according to www.clinicaltrials.gov. The tumor types included breast cancer, lung cancer and gastrointestinal cancer, among others, but not thyroid cancer. We look forward to the publication of additional findings on the efficacy of IORT for the treatment of thyroid cancer.
